# Higher Risk of Chronic Hepatitis E Virus Infection in Patients with Human Immunodeficiency Virus-1: An Iranian Cross-sectional Study

**DOI:** 10.30699/IJP.2023.551657.2870

**Published:** 2023-06-20

**Authors:** Mohammad Javad Zahedi, Sara Shafieipour, Mohammad Mahdi Hayatbakhsh Abassi, Nader Pourjamali, Mohsen Nakhaie, Javad Charostad, Mohammad Rezaei Zadeh Rukerd, Mohammad Mehdi Lashkarizadeh, Fatemeh Karami Robati, Azam Dehghani, Yunes Jahani, Seyed Ali Mohammad Arabzadeh, Hamid Reza Mollaei, Ebrahim Ranjbar

**Affiliations:** 1 *Gastroenterology and Hepatology Research Center, Institute of Basic and Clinical Physiology Sciences, Kerman University of Medical Sciences, Kerman, Iran*; 2 *Physiology Research Center, Institute of Neuropharmacology, Kerman University of Medical Sciences, Kerman, Iran*; 3 *Cardiovascular Research Center, Department of Cardiology, Institute of Basic and Clinical Physiology Sciences, Kerman University of Medical Sciences, Kerman, Iran*; 4 *Endocrinology and Metabolism Research Center, Institute of Basic and Clinical Physiology Sciences, Kerman University of Medical Sciences, Kerman, Iran*; 5 *Department of Microbiology, Shahid Sadoghi University of Medical Science, Yazd, Iran*; 6 *Colorectal Research Center, Shiraz University of Medical Sciences, Shiraz, Iran*; 7 *Clinical Research Development Unit, Afzalipour Hospital, Kerman University of Medical Sciences, Kerman, Iran*; 8 *Department of Epidemiology and Biostatistics, School of Public Health, Kerman University of Medical Sciences, Kerman, Iran*; 9 *Department of Medical Microbiology, Kerman University of Medical Sciences, Kerman, Iran*; 10 *Behavioral Disease Counseling Center, Kerman University of Medical Sciences, Kerman, Iran*

**Keywords:** Chronic Infection, Hepatitis E virus, HIV-1, Iran, RT-nested PCR

## Abstract

**Background & Objective:**

Occurrence of Hepatitis E Virus (HEV) infection may be common in Human Immunodeficiency Virus (HIV-1) patients and may lead to chronic infection as well as cirrhosis. We intended to determine the incidence of HEV infection among HIV-1 patients compared to individuals without HIV-1 infection.

**Methods:**

In our cross-sectional study, 87 HIV-1-positive patients were compared to 93 healthy individuals in Kerman, Iran. Plasma and peripheral blood mononuclear cells (PBMCs) were obtained from all the participants. Plasma samples were evaluated for HEV IgM and IgG using the ELISA kit. Then, reverse transcriptase-nested polymerase chain reaction (RT-nested PCR) was used in RNA extractions from PBMCs to check for the presence of HEV RNA.

**Results:**

Among the subjects examined in our study, 61 (70.1%) and 71 (77.4%) out of patients with HIV-1 infection and healthy individuals were male, respectively. The average ages of patients with HIV-1 and the control group were 40.2 years and 39.9 years, respectively. No discernible differences were found between the two groups based on IgM and IgG seropositivity against the HEV. However, HEV-RNA was found in 8% of patients with HIV-1 and 1.1% of HIV-1-negative individuals (P=0.03). There was also an association between the HEV genome and anti-HEV and anti-HCV antibodies in HIV-1-positive patients (P=0.02 and P=0.014, respectively).

**Conclusion:**

HEV infection may be more common in HIV-1 patients and may develop a chronic infection in immunocompromised individuals. Molecular-based HEV diagnostic tests, including RT-PCR assays, should be performed in HIV-1 patients with unknown impaired liver function tests.

## Introduction

Hepatitis E virus (HEV) is a non-enveloped, single-stranded RNA virus in the family Hepeviridae. For the first time in 1978, this virus was discovered during an out­break in India (1, 2). Like the same hepatitis A virus, its transmission occurs in a fecal-oral way, through contaminated food or water (3). More than 20 million people, according to the World Health Organization (WHO), become infected with HEV every year, of which 3.3 million have clinical symptoms and 44,000 deaths (4). So based on WHO, HEV is the leading cause of acute viral hepatitis worldwide (4). Anti-HEV IgM antibodies can be identified at the stage of clinical onset (3-4 days after the beginning of jaundice) and remain obvious for 3-12 months in acute HEV infection. Anti-HEV IgG antibodies appear soon after IgM antibodies and can last for years (5).

 HEV is the leading cause of endemic and sporadic cases of viral-induced hepatitis in developing nations, including in some parts of Asia, Africa, and the Mediterranean, with poor hygiene conditions and sanitation standards (6, 7). So, As a result, HEV infection is the leading cause of acute hepatitis in adults in Asia, and the second leading reason in the Middle East after hepatitis B virus (HBV) infection (8). According to a meta-analysis study conducted in 2016, the prevalence of HEV in Iran was reported to be between 1 and 46% and an average of 10% (9).

Based on our previous knowledge, HEV infections were exclusively associated with self-limited acute disease. However, in 2008, Kamar* et al.* described chronic HEV infection in eight immunocompromised organ-transplant recipients for the first time (10). Subsequently, chronic HEV infection has been observed in a few human immunodeficiency virus (HIV)-infected patients in different parts of the world (4). Persistent carriage of HEV in immunocompromised patients was linked to a lack of immune recovery and may be accompanied by liver fibrosis or progression to cirrhosis (11, 12).

In HIV+ patients, co-infection with HEV may be overlooked because elevated liver enzymes are common in antiretroviral therapy. This increase in enzymes due to co-infection with HEV may be attributed to drug side effects and not follow-up for the patient (13). 

HEV prevalence in HIV-infected patients has been noted from <10% to >40% (4). Some studies have reported that HIV-positive patients are more likely to become infected with HEV than HIV-negative individuals. For example, according to Scotto* et al.*, the prevalence of anti-HEV within HIV-infected patients (6.7%) was reported to be more than 2 times higher in the general population (2.7%) (14). On the other hand, some studies found no differences in anti-HEV frequency between HIV-infected patients and non-HIV individuals (15). HEV infection in patients with HIV depends on factors such as geographical area and study population; in European countries, it has been reported from 0.95 to 26% percent (16). Moreover, it is indeterminate whether co-infected patients are predisposed to chronic infection and severe liver disease (17). 

Further studies show that HEV prevalence is higher in HIV-infected patients. Therefore, the vaccine can be given to prevent HEV infection, which can cause chronic hepatitis in immunocompromised patients, or set up a screening program for HIV-positive patients to diagnose co-infection with HEV. As reported in the case report study, if chronic HEV develops in HIV-positive patients, treatment with ribavirin and interferon alfa can rapidly normalize liver enzyme levels and, after a few months, the HEV load in the patient's serum and feces will be significantly reduced (18). It also increases CD4 count following treatment and reduces liver tissue's inflammation and fibrosis (18).

Therefore, according to the above points, we decided to investigate the prevalence of HEV infection in HIV-1-infected patients compared to healthy individuals by ELISA and RT-nested PCR methods in Kerman, the center of a large province in South-East Iran – A country with a slightly elevated HIV and HEV infection rate.

## Material and Methods

**Study Population and Patient Information**

From March to June 2018, a total of 87 HIV-1-infected patients were referred to the Kerman behavioral health center and included in our study. The control group was 93 healthy blood donors who tested negative for HIV-1, HBV, as well as hepatitis C virus (HCV). Demographic data from all patients and control groups were collected from their medical records and entered into a checklist. The Ethics Committee of Kerman University of Medical Sciences, Kerman, Iran, approved this study, under ethics number IR.KMU.REC.1393.397. The participants were verbally informed of the study's goal, and each signed informed consent.

**Inclusion and Exclusion Criteria**

The participants who filled the consent form and were aged ≥18 years old in patients, and with negative results of the serological and molecular tests for HIV in the healthy group were included. The participants with chronic liver disease, malignancy, particularly liver malignancy, as well as critical health conditions, were excluded.

**Collection and Preparation of the Specimens**

Of peripheral blood, 4 mL was collected from each patient and placed in two sterilized vacutainer EDTA blood collection tubes. The plasmas were separated from the blood samples by centrifugation at 2500 rpm for 10 min and preserved at -20°C until serological testing. PBMCs were isolated using Ficoll (Bahar-Afshan, Iran) by density gradient centrifugation, and Phosphate-buffered saline (PBS) was used to wash these cells three times. Cells were suspended in 200 μL of PBS solution and were preserved at -80°C as long as additional testing.

**Serological and Biochemical Tests**

The Anti-HEV antibody (anti-HEV IgM and IgG) levels were measured for all participants by using an enzyme-linked immunosorbent assay (ELISA); (no: EIA1813, Method: MTPL.96 wells; DRG co, Germany) in accordance with the instructions provided by the manufacturer. Anti-HEV IgM and IgG sensitivity and specificity were 100%. Additionally, liver function tests (LFT), such as aspartate aminotransferase (AST) and alanine aminotransferase (ALT), were tested on all HIV-1- infected patients. To check the status of HIV-1-positive patients for anti-HBV and anti-HCV antibodies, the medical records of HIV-1 patients were also examined at the Center for Behavioral Diseases.

**Genomic RNA Preparation and Detection of HEV RNA by RT-nested PCR**

According to the kit instruction, viral RNA from PBMC HIV-1 positive and negative samples were extracted using the High Pure Viral Nucleic Acid Kit (Roche Applied Science, Germany). Amplification of the human β-globin gene with PCO3 (ACACAACTGTGTTCACTAGC )/PCO4 (CAACTTCATCCACGTTCACC) primers was used to control the extracted genome's quality based on previous studies (19). The purity and concentration of viral RNA were measured by a NanoDrop spectrophotometer (Thermo, USA). In the next step, 0.5 μg of extracted RNA template was reverse-transcribed to cDNA in a final volume of approximately 20 µL, using a cDNA synthesis kit (Yekta Tajhiz, Iran) based on the manufacturer’s instructions. The cDNA was prepared and stored at -80°C up to additional analysis.

To detect the HEV genome, the HEV ORF2 genome sequence was amplified by RT-nested PCR with specific previous primers (20). The sequences of the outer primers were 5′ -GAG GCA GGC ACA ACT AAA GC- 3′ and 5′ -AAG AAG GGG GGC ACA AG- 3′, and The sequences of the inner primers were 5′ -GCA CCG GGT CGC TAT TTC- 3′ and 5′ -TGA AGC TCA GCG ACA GTA GA- 3′.

Both rounds of PCR were performed in a total reaction volume of 25 µL, which includes 1X PCR buffer (Tris-HCl, pH 8.3), 2 μL of cDNA/PCR product, 1.5 mM MgCl2, 20 mM KCl, 0.1 mM deoxynucleotide triphosphates (dNTPs), 20 pmol of each primer, and 1 U of Ampliqon Taq DNA Polymerase.

The PCR cycling protocol included 1 min at 94°C, followed by 2 min at 60°C, and 2 min at 72°C for 35 cycles in the first PCR, followed by a ﬁnal extension at 72°C for 8 min. In the second PCR, the reaction is performed using 2 μl from the product of the ﬁrst PCR product along with the internal primers and the same thermal cycling protocol as in the first round.

Finally, the second PCR product is electrophoresed and runs on 2% agarose gel, and 216 bp bands indicate the presence of the HEV genome. In each HEV RT-nested PCR run, positive and negative control were included.

**Statistical Analysis**

Frequency and percentage were used for categorizing qualitative variables. Pearson’s Chi-square test (X²) and logistic regression analysis were used to compare LFT and the results of virologic tests between groups. SPSS software version 22 was used for data analysis. GraphPad Prism, version 8.0.2 (Graphpad Software, Inc.), was also used to draw graphs. P-value<0.05 was considered statistically significant. 

## Results

**Clinical Parameters**

In our study, there were a total of 93 healthy blood donors in addition to 87 HIV-1 infected patients. Male individuals made up 61 (70.1%) of those who tested positive for HIV-1 and 71 (77.4%) of those who tested negative for the virus. The mean age of the HIV-1 positive group was 40.2 and for the control group was 39.9 years old. There were no statistically significant differences in age or gender between the two groups (*P=*0.811 and *P*=0.265, respectively). Of the 87 HIV-1-positive individuals, 61 and 71 had an abnormal elevation in AST and ALT enzymes (>40 units/L), respectively. 

**Serological Tests**

HEV diagnostic markers in participants were 4.6% in the patient's group and 7.5% in the healthy group for anti-HEV IgM, 18.4% in the patient's group, and 21.5% in the healthy group for anti-HEV IgG, which was not statistically significant (*P=*0.412 and *P*=0.630, respectively) (Figures 2 and 3). Among HIV-1-positive patients, only one patient tested positive for anti-HBV and anti-HEV antibodies, and 5 were positive for anti-HBV antibodies but negative for anti-HEV antibodies (*P*=0.999). While 11 HIV-1-positive patients were positive for anti-HCV and anti-HEV antibodies, 36 were positive for anti-HCV antibodies and negative for anti-HEV antibodies (*P*=0.191) (Table 1).

**Table 1:** Results of anti HEV antibody with anti HBV and anti HCV antibodies in HIV-1 positive patients

		Anti HEV antibody		P-value	OR(CI)
		Positive	Negative		
Anti HBV antibody	Positive	1 (6.3%)	5 (7%)	0.999	0.88 (0.8-0.1)
	Negative	15 (93.8)	66 (93)		
Anti HCV antibody	Positive	11 (68.8%)	36 (50.7%)	0.191	2.13 (6.8-0.67)
	Negative	5 (31.3)	35 (49.3)		

The frequency (percentage) of the data was shown based on the chi-square test.

**Fig. 1:**
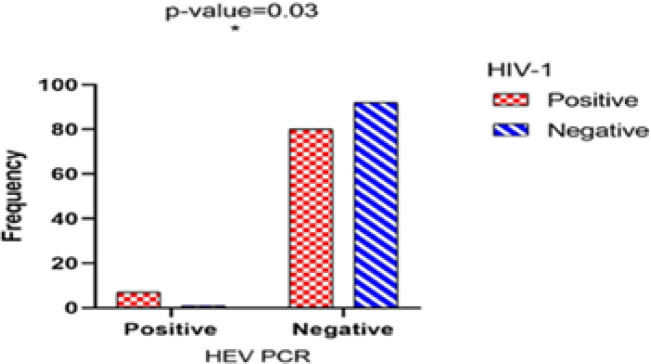
Results of the HEV PCR test in HIV-1 positive and negative participants

**Fig. 2:**
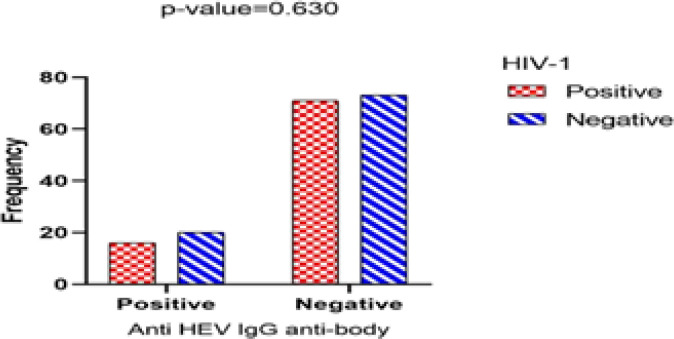
Results of the anti-HEV IgG test in HIV-1 positive and negative participants

**Fig. 3:**
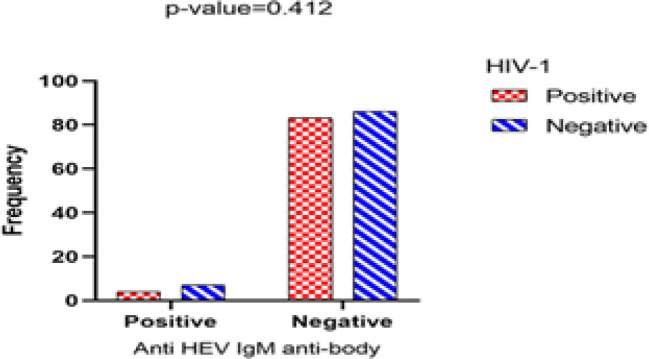
Results of the anti-HEV IgM test in based on HIV-1 status

**Results**

The HEV genome was found among 2.2% of blood samples (8 out of 180). Molecular evidence presented significantly higher HEV RNA positivity in 8%(7 out of 87) HIV-1+ samples as compared with 1% (1 out of 93) in HIV-1- samples (*P*=0.03) (Figure 1).

By comparing the frequency of HEV genome-positive cases with anti-HEV, anti-HBV, and anti-HCV antibodies in both patient and healthy groups, the statistical correlation between HEV genome-positive cases and anti-HEV IgG and also between HEV genome-positive cases and anti-HCV Ab in HIV-1-positive patients were observed (*P*=0.02 and *P*=0.014, respectively)( Table 2).

In addition, in the group of HIV-1-positive patients, 83.4% of HEV genome-positive patients had a CD4+ count <150mm3, 83.4% of cases were alcohol consumers >12 drinks a week and all of the patients had oral sex (Table 2). 

Data analysis demonstrated no statistical difference between HEV PCR positivity and abnormal elevation of liver enzymes ALT and AST in HIV-1-positive patients (*P*=0.347 and *P*=0.77, respectively) (Table 2).

**Table 2:** Virologic, CD4+ count and some behavior characteristics in the HIV-1 patients with HEV PCR positive and negative

		HEV PCR		P-value	OR(CI)
		positive	negative		
HEV Ab	Positive	4 (25)	12 (75)	0.02	7.55 (38-1.49)
	Negative	3 (4.2)	68 (95.8)		
HCV Ab	Positive	4 (57.1)	40 (50)	0.014	8 (1.3-50)
	Negative	3 (42.9)	40 (50)		
HBs Ag	Positive	3 (42.9)	32 (40)	0.882	0.998 (0.1-9)
	Negative	4 (57.1)	48 (60)		
AST	<40	6 (85.7)	55 (68.8)	0.347	2.7 (23.8-0.3)
	>40	1 (14.3)	25 (31.3)		
ALT	<40	6 (85.7)	65 (81.3)	0.77	1.3 (12.3-0.1)
	>40	1 (14.3)	15 (18.8)		
CD4+ count	>150mm3	1 (16.6)	11 (13.7)	0.27	2.9 (0.4-24.1)
	<150mm3	6 (83.4)	69 (86.3)		
Oral sex	Yes	7 (100)	79 (98.8)	0.812	1.02 (0.1-12)
	No	0 (0)	1 (1.2)		
Alcohol>12 drink week	Yes	6 (83.4)	76 (95)	0.501	2.1 (0.21-22)
	No	1 (16.6)	4 (5)		
Opium misuser	Yes	6 (83.4)	75 (93.8)	0.405	2.5 (0.25-25)
	No	1 (16.6)	5 (6.2)		

The frequency (percentage) of the data shown is based on the Chi-square test.

## Discussion

The susceptibility of HIV patients to HEV infection has remained questionable. Results from some studies show higher HEV/HIV co-infection, especially in those who are more immunodeficient and live in endemic regions (21). However, the data demonstrating the epidemiology of HEV in immunocompromised patients is unclear and needs further studies (22). Therefore, the present study was undertaken to investigate the prevalence of antibodies and the presence of the HEV genome in HIV-positive patients compared to the healthy individuals.

In the first step, we evaluated the prevalence of serologic viral hepatitis markers in the all participants. Our data highlighted that there is no correlation between the presence of HEV antibodies and HIV-1 positivity (Figure 2,3). Generally, the seroprevalence of IgG anti-HEV in HIV-positive patients varies in different parts of the world, with more than 40% reported in African and Asian countries, between 10%-20% in European countries, and less than 10% in the Americas and Oceania (23). several serologic findings from different geographic regions present discrepant results. For example, in a study performed on 158 HIV-positive cases in Shiraz (southern Iran), by Joulaei* et al.*, 16.4% HEV seropositivity was found in the patients (24). Another study from Spain by Vázquez-Morón* et al.* reported the prevalence of IgM anti-HEV Ab and IgG anti-HEV Ab as 27.3% and 57.1 %, respectively (*P*<0.001), in HIV monoinfected patients (16). However, in contrast to those studies, In Mateos-Lindemann* et al.* study of 448 HIV-positive patients in Spain, anti-HEV IgG prevalence was 10.4% compared to 2.9% in healthy blood donors, and there was no difference in seroprevalence between the two groups (*P*>0.05) (25). Although the prevalence of HEV antibodies in our study was slightly higher among HIV-positive patients than in other studies, compared to our control group, We discovered that the prevalence of HEV was relatively high in our region. Also, This inconsistency of the results of studies indicates the different epidemiology of these two diseases in each region.

In the next step, through assessing the presence of HEV genome-positive cases in both patient and healthy groups, the association between chronic HEV infection and HIV-1 positivity was shown. Therefore, 7 out of 8 HEV genome-positive cases were in the patient group, and 1 was in the healthy group (7/87; *P*=0.03) (Table 2). The data obtained in our study is consistent with those of Salvio, which showed the significant of the RT-qPCR method in identification of HEV-RNA in HIV+ patients with low viral load in their serums and those in whome anti-HEV IgG and IgM could not be detected by ELISA (26). Although previous studies have mentioned that HEV infection never become chronic, and in some studies, cases of chronic HEV were not identified. This part of the study showed a higher prevalence of the HEV genome in PBMCs of HIV-positive patients than in HIV-1-uninfected individuals by RT-nested PCR. These results, along with reports of hepatitis E in immunocompromised patients such as organ transplant recipients and patients with non-Hodgkin lymphoma receiving immunosuppressive therapy, indicate the prevalence of chronic HEV infection in immunocompromised patients (10, 27).

Although in the current study, a comparison of the anti-HEV antibody level concerning anti-HCV and HBV antibodies did not show a statistically difference in HIV-1 + patients, there was a significant relationship between HEV PCR positivity and the presence of HCV Ab in HIV-1+ participants (*P*=0.014) (Table 2). It is worth noting that in HIV + patients co-infected with HCV, the footprint of HEV infection has been suggested in previous investigations. As in a study performed by López-Fabal* et al.*, the level of Anti-HEV IgG was evaluated in three groups including HIV-infected patients, HCV-infected patients, and co-infected HIV-HCV. The results reported were 12.5%, 23.5%, and 40%, respectively (28). In another study published by de Moraes* et al.*, the analysis showed viral agents-related cirrhosis (HCV, HBV, and HBV/HCV co-infection), especially HCV infection, were associated with the highest positivity rates of anti-HEV IgG and detection of HEV-RNA (29). 

Evidence from this part of the study, along with previous studies, shows a significant association between chronic HEV and chronic HCV, which can lead to an increase in liver enzymes, and the progression of the late stages of liver disease such as cirrhosis and cancer may be considerable. 

In the last step, the relationship between chronic HEV infection and other parameters, especially liver enzymes, was evaluated, but in none of them was a significant relationship observed. Similar to the results of this study in some studies, the level of liver enzymes (ALT and AST) in patients was not abnormal. There was no significant relationship found between the presence of HEV and the level of liver enzymes (30-32). However, in another study in 2017 in Nepal, HIV-positive patients infected with HEV had significantly higher liver enzyme (ALT and AST) levels (33). Here our failure may be related to the study sample size and the small number of HIV-1 patients with HEV RT-PCR positive results, suggesting that further studies without these limitations are needed.

However, the limitation of our study was that we did not follow the HEV-positive PCR individuals in HIV-1 patients because the persistence of HEV-RNA in plasma for more than 6 months, with or without the development of anti-HEV antibodies, has been defined as chronic HEV infection (34). However, the strength of our work was the performing of HEV RT-nested PCR for the diagnosis of HEV infection in all participants. While in most studies, HEV diagnosis was limited to serology alone, or PCR was done only in seropositive cases (16, 24, 25, 35-37).

Our findings suggest that serologic screening alone may be insufficient to diagnose HEV/HIV-1 co-infection because seroconversion may be late or not occur in immune-suppressed patients. (12) In addition, the sensitivity and specificity of serologic assays for HEV diagnosis are undetermined (31). Since HEV infection may be fulminate in the presence of underlying liver disease and may lead to chronic infection in immunosuppressed persons (11, 35), we propose that HEV infection should be considered, especially in HIV-1-infected patients with unexplained impaired LFT.

## Conclusion

In the present study, we displayed that HEV may be more prevalent in HIV-1 patients and may result in persistent infection. Moreover, we recommend that HEV diagnostic tests, including HEV-RNA assays, should be performed, especially in HIV-1 patients who have previously unidentified impaired liver function tests.

## Conflict of Interest

None to declare.

## Funding/Support

Non

## Ethics Approval

The study was approved by the Ethics Committee of Kerman University of Medical Sciences, kerman, Iran, under number IR.KMU.REC.1393.397.
